# Supplementary material for: Synthesis and Characterization of a Full-Length Infectious cDNA Clone of Tomato Mottle Mosaic Virus
Source: Viruses. 2021 Jun 1;13(6):1050. doi: 10.3390/v13061050 (PMC8229035; doi:10.3390/v13061050)
Supplement: Supplementary file 1 [file viruses-13-01050-s001.zip › Supplementary Materials.pdf]

Supplementary Materials

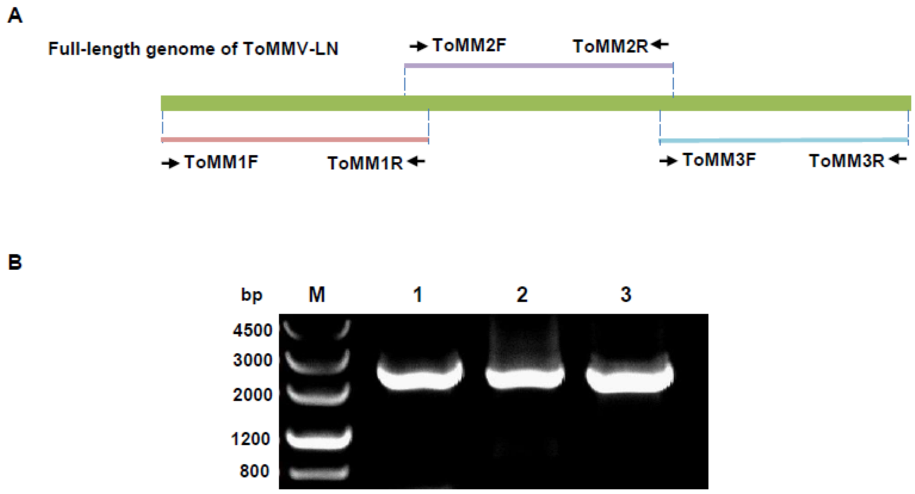

Figure S1. Three segments amplification of the full-length genome of ToMMV-LN. (A) Three fragments were designed to construct the full-length genome of ToMMV-LN. (B) Three fragments were amplified to construct the full-length genome of ToMMV-LN. M: DNA marker 3. Lane 1, 2 and 3: Amplification of first fragment about 2286 bps, 2310 bps and 2079 bps.

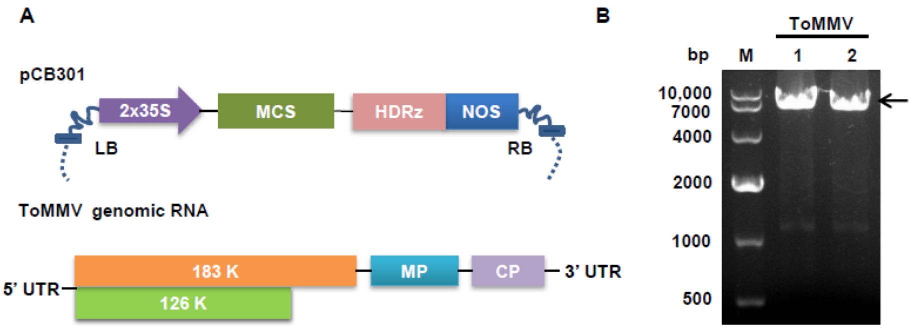

Figure S2. Amplification of the full-length ToMMV genome. (A) Schemes of the plant expression vector pCB301-2x35S-MCS-HDVRZ-NOS and ToMMV genomic RNA. 2x35S: CaMV 35S promoter. HDRz: hepatitis delta virus ribozyme. NOS: Nos terminator. LB: left border sequence. RB: Right border sequence. (B) Amplification of the full-length cDNA of ToMMV-LN. M: DL10,000 DNA Marker. Lanes 1-2: RT-PCR products of the full-length cDNA of ToMMV-LN.

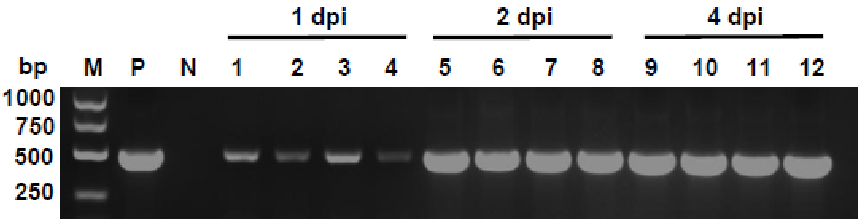

Figure S3. RT-PCR detection of ToMMV genomic RNA in argoinfiltrated leaves at 1, 2 and 4 dpi. M: DL5000 DNA Marker. P: positive control, pToMMV plasmid. N: negative control, healthy *N. benthamiana* plant. Lanes 1-4, 5-8 and 9-12: pToMMV-argoinfiltrated leaves at 1, 2 and 4 dpi.
